# Chemical Sensors and Imaging: Molecular, Materials, and Biological Platforms

**DOI:** 10.1021/acscentsci.3c01070

**Published:** 2023-09-18

**Authors:** Marco S. Messina, Christopher J. Chang

## ACS Virtual Issue on Sensing and Imaging

Perhaps nowhere in chemistry illustrates the proverb “seeing is believing” like the fields of sensing and imaging. From the invisible gases that make up the air we breathe, to the nanoscopic and microscopic molecules of life that make up the central dogma of DNA to RNA to proteins, to the metal ions and metabolites that shape cell behavior from firing of action potentials of neurons or triggering cell growth and death pathways in cancer and infectious diseases, sensors can make the invisible visible and help us understand and better our daily lives. In this virtual issue, we highlight innovative and important advances in sensing and imaging technologies in the chemistry published in *ACS Central Science* that make use of either small-molecule, biological, or materials probe platforms. These tools enable detection of chemistry across many length scales for fundamental biological discovery to devices for translational diagnostic applications in medicine and the environment.

## Small-Molecule Probe Platforms

Synthetic, small-molecule probe platforms continue to underpin a significant amount of biological discoveries using chemical sensors. Ample opportunities exist for innovation using archetypical small molecule fluorophores (e.g., fluoresceins, rhodamines, Si-rhodamines (SiR), etc.) to improve localization, enable ratiometric imaging, and enhance photophysical and molecular characteristics for implementation in super-resolution imaging technologies, such as stimulated emission depletion (STED) imaging and single molecule localization microscopy (SMLM). A key parameter in creating suitable rhodamine-based dyes for super-resolution imaging is to control the equilibrium between the closed lipophilic nonfluorescent lactone form and the open zwitterionic fluorescent form. Dyes optimal for super-resolution imaging should exhibit a spirocyclization equilibrium that lies predominately toward the lactone form. Along these lines, the Lavis group established quantitative design principles which dictate that the lactone-zwitterion equilibrium constant (*K*_L-Z_) is sufficient for the prediction of fluorogenicity.^1^ A dye with a low *K*_L-Z_ spends most of the time in its closed nonfluorescent form thereby greatly improving cell permeability but exhibits significant enhancements in absorption and fluorescence upon binding with a target of interest, characteristics which make an ideal dye for super-resolution imaging. After determining the *K*_L-Z_ of a large palette of rhodamine-based dyes, focusing on Janelia Fluor (JF) derivatives, they went on to develop JF_526_, which serves as a highly versatile dye that can be easily modified with an array of self-labeling tags (HaloTag, SNAP-tag ligands) or other targeting groups that enhance localization within the cell.^1^ Moving into the near-IR wavelength segment of the electromagnetic spectrum, a collaboration between the Bewesdorf and Schepartz laboratories introduced a photostable and near-IR-emitting dye, Yale_676sb_, for use in SMLM.^2^ Their work highlights a methodical approach to fluorophore design, where they improved upon the previous gold-standard SMLM dye, hydroxy-methyl Si-rhodamine (HMSiR), introduced by Urano.^3^ A panel of indoline, julolidine, and tetrahydroquinoline SiR derivatives exhibits red-shifted excitation/emission profiles and enhanced quantum yields due to restriction of twisted intramolecular charge transfer processes. Further improvements to the photophysical and spirocyclization equilibrium properties of the dyes were made via modification of the heterocyclic amines with electron-withdrawing alkyl fluorine substituents. They then went on to demonstrate the utility of the tetrahydroquinoline SiR derivative, Yale_676sb_, in tandem with HMSiR for the two-color, live-cell SMLM imaging of both the endoplasmic reticulum and mitochondrial membrane simultaneously.^2^

In the aforementioned studies, fluorophore localization was promoted through conjugation of small-molecule organelle targeting groups to the dye scaffolds or via genetically encoded self-labeling tags. However, biorthogonal chemistries such as copper-catalyzed or strain promoted azide–alkyne cycloaddition, and Staudinger and tetrazine ligation, among others, remain popular alternatives for enhancing dye localization.^4−6^ This situation is due in large part to the avoidance of specialized genetic modification techniques and the small size of molecular payloads compared to protein tags, which minimize perturbations within the biological systems being investigated. The Herten and Wombacher teams have introduced a panel of SiR dyes harboring tetrazine handles, labeled “Heidelberg Dyes” (HDyes), that are amenable to both STED and SMLM techniques.^7^ Anchoring the tetrazine scaffold near the SiR core enabled efficient fluorescence quenching of the free dye with large fluorescence enhancements upon inverse electron demand Diels–Alder (DA_inv_) labeling of alkyne-labeled biomolecules. The utility of these HDyes was showcased in high-resolution live-cell imaging of both intra- and extracellular targets modified with alkyne tags via unnatural amino acid incorporation.

Voltage-sensitive fluorescent reporters (VFdyes) are vital for measuring membrane potential in neurons and cardiomyocytes. However, photobleaching of traditional reporters makes it challenging to perform prolonged imaging experiments, and phototoxicity results in alterations to neuronal physiology. To bypass these limitations, Miller and co-workers reported a creative approach wherein they appended a cyclooctatetraene (COT) scaffold to a VFdye.^8^ The COT group acts as a triplet state quencher (TSQ) to reduce both photobleaching of the dye and phototoxicity toward neurons and cardiomyocytes. Notably, this single molecule TSQ tethering approach presents an attractive alternative toward nondisruptive membrane potential imaging, which previously relied on the exogenous addition of photoprotectant cocktails in millimolar concentrations.

The development of small-molecule sensors able to measure biorelevant analytes or enzyme activity through activity-based sensing strategies remains an area of intense research interest.^9,10^ Measurement and tracking of amino acid pools in live mammalian cells is one such example, as amino acid regulation is pivotal for maintaining cellular homeostasis. Glass, Lin, and co-workers developed a turn-on fluorescent sensor, NS560, that can visualize 18 of the 20 proteinogenic amino acid residues in mammalian cell models, with no response observed for proline and tryptophan.^11^ NS560 exhibits unprecedented fluorescence enhancements upon amino acid binding, with an ∼800-fold increase for glutamate. Deploying NS560 in live mammalian cell models, they visualized changes in the localization of amino acid pools upon pharmacological intervention via fluorescence microscopy. Notably, they observed that chloroquine treatment results in the accumulation of amino acids in cellular foci, a result that was not observed with other autophagy inhibitors. Subsequent chemical proteomics experiments identified Cathepsin L as the primary chloroquine target that led to amino acid accumulation, thus resulting in the discovery of a new mechanism of action for chloroquine.^11^ Kim and co-workers exploited a hypoxia-responsive enamine *N*-oxide molecular scaffold in both therapeutic and diagnostic applications. The enamine *N*-oxide motif plays a dual-function as it can undergo 2e^–^ reduction under hypoxic conditions to release an attached payload (i.e., fluorophore or drug) while simultaneously generating an electrophilic species amenable to modification by nucleophilic amino acid residues on proximal biomolecules.^12^ By installing the enamine *N*-oxide trigger onto an array of distinct synthetic molecules, the Kim laboratory enabled both *in vitro* and *in vivo* cell and tumor imaging and prodrugs of the pan-kinase inhibitor staurosporine.

Another important area of research covered in this virtual issue deals with the creation of sensors that enable the noninvasive surveillance of therapeutic effectiveness. Rao and colleagues provided an elegant example of this approach through their granzyme B sensitive nanoaggregation tracer (G-SNAT) platform, which measures checkpoint blockade and CAR T-cell therapy effectiveness.^13^ G-SNAT measures the activity of granzyme B (gzmB), a key enzyme involved in the granule-mediated cytotoxicity mechanism deployed by immune effectors. Cy5 labeled G-SNAT proved successful in measuring gzmB activity in CAR T treated lymphoma tumor-bearing mice. Moreover, G-SNAT was used in combination with bioluminescence imaging to measure the effectiveness of combination therapies, notably anti-PD-1 and anti-CTLA-4, in colorectal tumor-bearing transgenic mouse models expressing firefly luciferase via multimodal imaging. The longitudinal and multimodal *in vivo* imaging provided by the G-SNAT platform revealed insights into immune cell trafficking and tumor infiltration.^13^

Also highlighted in this virtual issue is a compelling Outlook by Freedman and co-workers, where they examine opportunities for sensing through the lens of the quantum world.^14^ The case is presented for increased attention into the development of molecular quantum sensors that combine the modularity and tunability of molecular sensors with the incredible sensitivity of quantum sensors.

## Biological Probe Platforms

Central to biological probe platforms is the use of specially tailored biomolecules (e.g., antibodies, peptides, or enzymes) typically used to enhance targeting specificity or to catalyze reactions in conjunction with synthetic small molecule or protein-based reporters that promote a visual response. In one example of this approach, Lin, Su, and colleagues engineered the ATP-independent luciferase, NanoLuc, to develop kinase-modulated bioluminescence indicators (KiMBIs) for real-time, noninvasive, *in vivo* imaging of Ras-ERK pathway inhibitor activity in the brain.^15^ Their strategy addresses long-standing challenges inherent to the measurement of inhibitor activity of drugs targeting the brain and establishes the first example of a bioluminescent readout for Ras-Raf-MEK-ERK pathway inhibition.

Platforms based on genetically encoded tags, such as HaloTag, remain a popular approach for controlling fluorophore localization. Along these lines, Straková, López-Andarias, Matile, and co-workers created “HaloFlipper” probes that consist of twistable dithienothiophene groups on one end and a chloroalkane tail on the opposite end that serves as a HaloTag substrate for targeting membranes of interest (MOI).^16^ They demonstrated the generalizability of their HaloFlipper platform for measuring membrane tension via live cell fluorescence lifetime imaging microscopy (FLIM) across a variety of organellar membranes, including those of the mitochondria, endoplasmic reticulum, peroxisomes, endolysosomes, and the Golgi apparatus.

Beyond enzyme-based methods for targeting or catalytic signal generation, single-stranded catalytic DNA molecules known as DNAzymes offer an attractive route toward selective monitoring of transient monovalent metal ions in biological settings. Group 1 metals, particularly lithium (Li^+^), remain challenging to track via fluorescence microscopy. Lithium remains the drug of choice for treating bipolar disorder (BD), but its mechanism of action is poorly understood, and many patients do not respond to this therapeutic intervention. Lu and co-workers created a novel Li^+^-specific DNAzyme fluorescent sensor through an *in vitro* selection process.^17^ The DNAzyme exhibits >100-fold selectivity of Li^+^ over other biorelevant metal ions and sensitivity that enables measurement of Li^+^ down to 1 mM concentrations in live cells. This team further applied their sensors to monitor Li^+^ pools in a human-induced pluripotent stem cell (iPSC)-based model. Interestingly upon Li^+^ treatment, they observed increased Li^+^ accumulation in differentiated neurons derived from BD patients compared with controls from healthy patients, but similar trends were not observed in neuronal progenitor cells.

Turn-on type fluorescence sensors containing protease cleavable peptide handles also offer an attractive avenue for targeted imaging, but there remains a need for ratiometric alternatives that benefit from internal normalization and inertness toward changes in pH and polarity. Bogyo, Guerra, and co-workers created ratiometric contrast agents to aid in the surgical removal of cancerous tumors.^18^ They showed that the cathepsin cleavable probe 6QC and the AND-gated dual responsive probe Death-Cat-2 could be converted into ratiometric derivatives through modification with both Cy5 and Cy7 dyes, thereby producing 6QC-RATIO and Death-Cat-RATIO reporters, respectively. These new sensors exhibited superior sensitivity over previously reported compounds enabling the detection of small distal tumors in mouse models that other probes missed. Additionally, they created a new imaging device for the real-time concurrent recording of both Cy5 and Cy7 emissions for use during surgical procedures.^18^

Raman bioimaging is currently undergoing a renaissance given advances made in instrumentation and methodology. The Wei group is among those at the forefront of Raman bioimaging and reported stimulated Raman scattering (SRS) to study polyglutamine protein aggregates in live cells.^19^ Polyglutamine protein aggregates are implicated in an array of neurodegenerative disorders, such as Huntington’s disease. By deuterium labeling glutamine residues in mutant Huntingtin (mHtt) exon 1 proteins and studying their aggregation in live cells via ratiometric SRS imaging, they determined the absolute concentrations for mHtt vs non-mHtt proteins within aggregates, thereby unearthing a dependence of protein composition on aggregation size. They also made discoveries which contrasted with previous reports regarding the structure and aggregation density of mHtt aggregates, thus highlighting distinct advantages of SRS imaging over fluorescence techniques for obtaining conformational and structural information *in vitro*.

The SARS-CoV-2 pandemic has prompted the development of novel sensing platforms that enable rapid and sensitive viral detection. The quick spread of the SARS-CoV-2 virus, coupled with the long wait times and technical requirements of testing using reverse transcription-polymerase chain reaction (RT-PCR)-based techniques, necessitates the creation of portable and easy-to-use diagnostic tests. Rodriguez-Manzano and co-workers tackled this challenge by introducing a point-of-care diagnostic test that produces results in less than 20 min.^20^ The platform is based on loop-mediated isothermal amplification (RT-LAMP) coupled with semiconductor technology developed in their laboratories which measures pH changes during nucleic acid amplification. The authors went on to create easy-to-use devices that could be coupled with a smart phone application. To prepare for the emergent global health threats posed by viral mutations, the Amaro and Freeman laboratories utilized a biomimetic approach to create a lateral flow assay for detecting SARS-CoV-2 and its variants termed *GlycoGrip*.^21^ They employed computational approaches to gain mechanistic insight into binding interactions between the SARS-CoV-2 spike protein and glycocalyx components, identifying six novel binding sites. These results were used to optimize the *GlycoGrip* assay which detected SARS-CoV-2 and spike variants down to low μg/mL concentrations in human patient saliva samples. The use of glycopolymers found in the glycocalyx enhances antigen binding as a result of multivalent interactions and can be easily tuned to capture mutated strains, thus affording a highly generalizable and inexpensive assay. Merkx and co-workers developed an elegant strategy for the detection of SARS-CoV-2 that uses a CRISPR-Cas-9-based split luciferase complementation strategy.^22^ The platform they developed, named LUNAS (Luminescent Nucleic Acid Sensor), uses reverse transcription-recombinase polymerase amplification (RT-RPA) for the amplification of target nucleic acid. The target nucleic acid is identified by a pair of dCAS9-based probes which mediate the complementation of split NanoLuc luciferase and gives rise to a blue luminescent readout. A calibrator luciferase enables ratiometric reporting, and the platform as a whole was demonstrated to be easy-to-use with a simple, highly sensitive, readout that could be recorded on a digital camera.^22^

Bacterial infections such as tuberculosis remain a leading cause of death worldwide, disproportionately affecting under-resourced areas. Access to sensitive, quick, and affordable diagnostic tests is imperative for mitigating the spread of infection. Bogyo and colleagues developed a fast, luminescent, and affordable sensor of Hip1 (FLASH).^23^ The FLASH probe acts as a substrate for Hip1, a *Mycobacterium tuberculosis* (*Mtb*) protease, and produces a chemiluminescent readout upon proteolytic cleavage. Notably, FLASH exhibits a limit of detection down to 15,000 cells in human sputum and is highly selective for *Mtb* over other nontuberculous mycobacteria. Additionally, this platform was proficient in differentiating live from dead bacteria, thus enabling FLASH to be leveraged for the measurement of antibiotic killing of *Mtb* and offering a faster alternative over traditional approaches for determining drug susceptibility.

## Materials Probe Platforms

The rapid growth of research into porous materials such as metal–organic frameworks (MOFs) has spawned a flurry of activity in using such platforms for sensor development.^24^ With the increasing dangers of pollution and climate change, gas sensing is at the forefront of ongoing sensor research in this area. Against this backdrop, Dincă and co-workers designed chemiresistors incorporating 2D MOFs based on hydrated Cu_3_(hexaiminobenzene)_2_ frameworks for the detection of CO_2_ under ambient conditions.^25^ Notably, they were able to detect CO_2_ down to 400 ppm across a broad range of relative humidity (10–80%), addressing a limitation of traditional devices that exhibit diminished performance depending on humidity level. Nitrogen dioxide (NO_2_) represents another atmospheric pollutant prompting interest in sensor development. Lee and colleagues created the first reversible NO_2_ MOF-based hybrid sensors composed of Cu_3_(HHTP)_2_ and Fe_2_O_3_ nanoparticles.^26^ These materials exhibit exceptional selectivity and sensitivity toward NO_2_ detection, which can be reversed via visible light irradiation, thereby releasing the captured NO_2_. The expanding roles of MOFs as materials in devices necessitates the creation of improved synthetic techniques. Bloch, Rosenthal, and their teams reported the rapid syntheses of four iron-based MOFs via the direct electrochemical oxidation of solubilized Fe^2+^ under ambient conditions.^27^ Their method for accessing MOFs overcomes challenges associated with indirect electrochemical techniques, such as unwanted coreduction of metal ions, and obviates the need for high temperatures, pressures, and long reaction times of traditional synthetic procedures. The high level of control inherent to this methodology enabled patterning of high-quality Fe-MIL-101 on carboxy-modified ITO electrodes.

Polyoxometalates (POMs) represent an important class of compounds with which to formulate chemiresistors. Swager and colleagues reported an exceptional demonstration of this approach through the development of hydrogen sulfide (H_2_S) sensors based on single-walled carbon nanotubes (SWCNTs) incorporating platinum POMs as highly oxidizing selectors.^28^ The POM-based sensors exhibit high selectivity for H_2_S at ppb-level detection limits and exceptional stability with only miniscule drops in response noted after two months of storage under ambient conditions. They went on to demonstrate the ease of device manufacturing via surface modification of a commercially available Kapton substrate, an aromatic polyimide film used in a variety of high-performance coatings. Also highlighted in this issue is another contribution from the Swager group in which they created activity-based sensors for ethylene that function via Wacker oxidation chemistry.^29^ They created a device composed of a catalytic mixture of palladium-based catalysts within a SWCNT network which enabled ethylene detection in ambient conditions down to ppb levels, even in the presence of other interferents. Excitingly, they were able to apply their device for monitoring flower senescence.

Mesoporous metal-oxides offer yet another attractive family of materials for sensing, catalysis, and storage applications owing to their high surface areas. Deng and colleagues introduced a facile synthesis of Pt nanoparticle-decorated Si-doped WO_3_ nanowires interwoven into 3D mesoporous superstructures (Pt/Si-WO_3_ NWIMSs) via multicomponent coassembly with amphiphilic polymer, polyoxometalate, and platinum precursors.^30^ These highly stable materials displayed excellent properties for the sensing of ethanol at low temperatures (100 °C) with high sensitivities and low limits of detection.

We also highlight articles in this special issue describing emergent imaging technologies and novel classes of nanomaterials. The Kenworthy group has established a high-throughput screen and image analysis pipeline that enables the identification of small molecule modulators of lipid raft formation.^31^ Cell membrane function is regulated, in part, by the formation of lipid rafts which are alternating assemblies of proteins and lipids within the plasma membrane that can cluster to form ordered domains. Key to their methodology is the fluorescence measurement of phase-separated giant plasma membrane vesicles (GPMVs) upon addition of a panel of small molecules over a broad concentration range while at constant temperature. Using their high-throughput screening methodology, they were able to validate existing small-molecule lipid raft modulators, while identifying completely new ones such as the protease inhibitor tosyl-l-lysyl-chloromethane hydrochloride (TLCK), which increased lipid raft formation. In the realm of materials characterization, Li, Zhang, and Han describe advances in four-dimensional scanning transmission electron microscopy (4D-STEM) to reveal atomic level structure in materials such as MOFs, hybrid halide perovskites, and supramolecular crystals, which are sensitive to traditional TEM-based analysis.^32^ Beyond highlighting advantages and applications of 4D-STEM, they also take a deep dive into data collection and processing methods which should aid both beginner and novice practitioners in the field. Finally, the rapid pace of materials science research results in the consistent generation of new materials. One such example presented in an Outlook article by Liu, Li, and Yang covers an emerging type of carbon-based nanomaterial referred to as carbon dots.^33^ They provide key insights into synthetic methods and highlight distinct differences of carbon dots from traditional carbon materials such as activated carbon, carbon fibers, and graphite. Additionally, the authors delve into applications and emergent research of carbon dots as sensors, catalysts, solar cells, and biomedical applications, among many others.

To close, we hope you enjoy reading this virtual issue on sensing and imaging as much as we have enjoyed organizing it. We believe it highlights the creativity and diversity of both early career and established researchers who are tackling the grandest challenges facing our world today through the development of innovative and impactful new sensing and imaging technologies. Chemistry is central to sensing and imaging!
